# Presenting the AI models in predicting the settlement of earth dams using the results of spatiotemporal clustering and k-means algorithm

**DOI:** 10.1038/s41598-024-60944-4

**Published:** 2024-05-03

**Authors:** Behrang Beiranvand, Taher Rajaee, Mehdi Komasi

**Affiliations:** 1 Civil Engineering, Water and Hydraulic Structures, University of Qom, Qom, Iran; 2 Department of Civil Engineering, University of Qom, Qom, Iran; 3 Department of Civil Engineering, University of Ayatollah Ozma Boroujerdi, Boroujerd, Iran

**Keywords:** Spatiotemporal clustering, Instrumentation, Plaxis 2D, Settlement, k-means algorithm, AI models, Hydrology, Engineering

## Abstract

In this work, the results of instrumentation over 8 years, including the phases of construction, first impounding, and operation, have been used to analyze the location of the Eyvashan Dam settlement. Mohr–Coulomb behavioral model and numerical model of Plaxis 2D software were used to verify the monitoring results. The results demonstrated that settlement of the dam has increased in the dam's core since the beginning of construction, and they eventually stabilized during the operation phase. After the completion of the construction phase, the maximum settlement of the dam core was recorded as 809 mm, which is equivalent to 1.2% of the height of the dam at the middle level. Also, an approach to interpreting the settlement behavior of earth dams has been presented that is based on spatiotemporal clustering. Also, RF, MARS, and GMDH models were created based on a proposed scenario to predict settlement using points located in a cluster. Therefore, the settlement location of the studied dam was determined using the results of the k-means clustering algorithm in the aforementioned AI models. The high accuracy of the results of the proposed method confirms the proper performance of using AI models in predicting and diagnosing the settlement of earthen dams using the results of k-means spatiotemporal clustering algorithm. The evaluation of the models shows that the ENN model is a more suitable and efficient tool in this field and can be useful in monitoring the settlement of earth dams.

## Introduction

In general, the safety of dams is not only related to design and construction, but also depends on the behavior and performance of instrumentation in the phases of construction, impounding, and operation, as well as regular servicing and monitoring throughout the life of the dam^1^. Monitoring settlement in earth dams has been the subject of many studies since the use of instrumentation data and comparison with numerical models^2^. Shi et al.^3^, presented a new modeling approach to provide an improved and reasonable statistical model for quantitative analysis of monitoring data. Nowadays, it is popular to monitor the deformation of CFRD dams during different periods using improved models and the results of instrumentations. Panel data has become a popular method for predicting deformation in many areas of civil engineering and geotechnics. Hu and Ma^4^, developed a method to anticipate the regional deterioration of arch dams by utilizing hierarchical clustering and panel data. In this model, instrumentation monitoring data from a real dam has been used for validation. Li et al.^5^, proposed a multiple monitoring points (MMP) model to predict and integrate spatiotemporal data regarding missing and insufficient data in faulty settlement sensors at rockfill dams. Cao et al.^6^, proposed a regional prediction model using the similarity distance index as a partitioning method for spatiotemporal data differences by fast search and finding of density peaks (CFSFDP), and spatiotemporal integrated autoregressive moving average model (STARIMA). By finding the similarity features of the deformation, the degree of similarity of the deformation is determined, and then using CFSFDP, the zones of the monitoring points with the same correlation value and deformation are clustered. In this study, the calculation of the integral distance between the measurement points and the rapid partitioning of the deformation monitoring points of the super arch dam was used. The STARIMA calculation framework creates a regional spatiotemporal analysis model of deformation that predicts the simultaneous movement of measurement points in the partition using CFSFDP rapid partitioning results. Deformation information in both time dimension and section can be found in the sequence of dam deformation and settlement at all monitoring points of the instrumentation. The dam's deformation status can be depicted in real time using spatiotemporal data from deformation monitoring data^7,8^. Wan and Doherty^9^ proposed a method based on data, which utilizes panel machine learning techniques and data regression, to estimate embankment settlement for multi-stage construction. Shi et al. ^10^, the deformation of concrete dams was mapped using the variable intercept panel method. Shao et al.^11^, Segmenting the deformation areas for monitoring points was done using the panel data model. Chen et al.^12^, the settlement monitoring data of concrete dams were presented through a spatiotemporal clustering method that can divide settlement areas into different periods. Measurement of similarity between the dam settlement characteristics can be enhanced by using panel data clustering, which is a suitable model for predicting in different single and hybrid models. Cai et al.^13^ used models MARS and GMDH to predict the deformation of earth dams in earthquake conditions. Using preliminary analysis, they developed intelligent models to predict the slope deformations. Najafzadeh^14^ used soft computing models including the M5 algorithm, group data processing (GMDH), and gene expression programming (GEP) to predict the behavior of Shahid Kazemi Bukan Dam. The results of their study showed that the GMDH model shows higher accuracy than the GEP and M5 models in predicting and monitoring dams. Ziggah et al.^15^ in the study of dam monitoring, analyzed the problem of dam seepage using the GMDH model and piezometric water level as an important parameter. Beiranvand et al.^16^, used k-means clustering and two-stage clustering algorithms to determine the displacement location of earth dams. The results of their study showed that the proposed method of detecting settlement areas can be a reliable method for monitoring the settlement of earth dams.

The purpose of this article is to create an approach for spatiotemporal clustering that can be used to monitor dam settlement. Therefore, the monitoring results of instruments and numerical models are used to calculate the maximum amount of settlement and location for the Eyvashan Dam. Also, the RF, MARS, and GMDH models have been used in the form of a panel data model to determine the location of the settlement at different cross-sections and levels, as well as to analyze the reliability and efficiency of the k-means cluster algorithm.

## Methodology

### Monitoring model of Eyvashan Dam settlement using panel data

**Figure 1:**
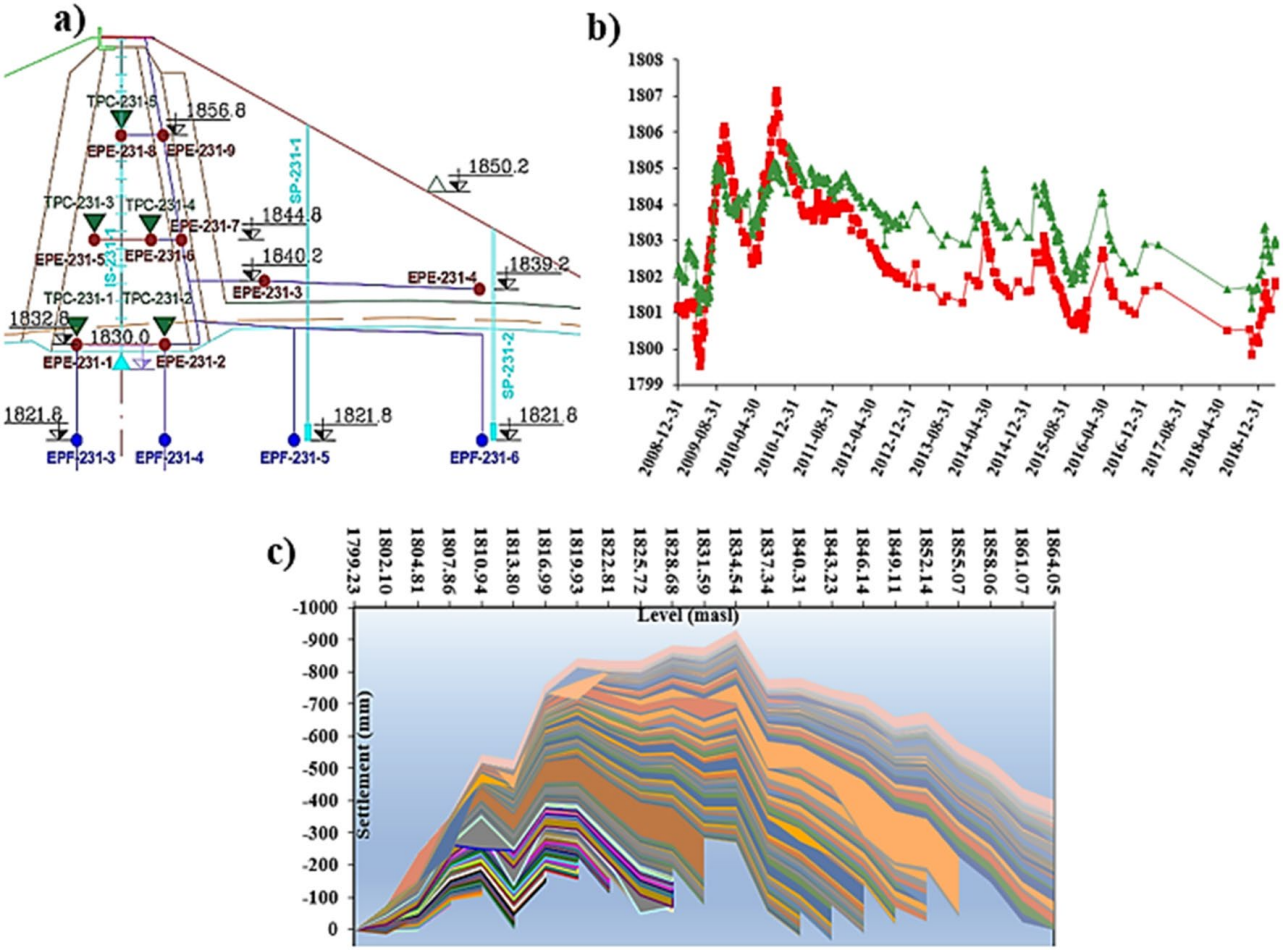
Schematic of dam settlement data: (**a**) cross-section data, (**b**) time series data, (**c**) panel data.

In the panel data regression model, regression analysis is performed in both spatiotemporal dimensions as part of a multivariate analysis. Traditional dam instrumentation analysis focuses on the time domain characteristics of individual monitoring points but neglects spatial information. By utilizing integrated panel data modeling instead of single-point models, model validity and accuracy can be enhanced by obtaining more dynamic information and experimental results. The location of monitoring points is displayed in cross-sectional data (Fig. 1a), and the temporal effects of cross-sectional data can be observed in time series data (Fig. 1b). As Fig. 1c indicates, time series and cross-sectional information are part of the two-dimensional panel data. Panel data combines cross-sectional data and time series, resulting in a correlation between monitoring points over time. The data is divided into two dimensions: one that pertains to various units at any given point in time, and another that pertains to time. Time series and cross-sectional data lack the efficiency and information of panel data, which has more variability and less collinearity. Combining cross-sectional and temporal data into panel data is a way to reduce collinearity. Panel data sets provide the ability to identify and measure effects that are not possible with pure cross-sectional data or pure time series. Different points typically have a large number of sensors placed to monitor the instrumentation of dams. The results of instrumentations that are placed in the vicinity and under the influence of environmental factors near certain areas (filters and drains) can lead to different results. Also, the monitoring time varies from the beginning of construction to the start of impounding and operation of the dam, however, the overall process of the instrumentation at different stages can be similar according to the upstream and downstream conditions.

The high similarity between tracking points is necessary for predicting missing data, and low correlation points should be screened. To eliminate outliers and confirm the consistency of monitoring data, data standardization is a suitable method. The monitoring points' similarity is influenced by the spatiotemporal conditions. The behavior of the dam both upstream and downstream is affected by instant consolidation during dam construction and pore pressure during impounding and operation. Dams' behavior has complex non-linear characteristics that change over time. The existing dataset on Eyvashan Dam reflects the dam's behavior during the construction phase from November 2008 to December 2013, the first impounding phase from December 2013 to May 2015, and operation from May 2015 to April 2019. Also, in terms of spatial, the behavior of the dam is different in the section and the whole structure, so the upstream areas of the dam that are affected by the reservoir water will have different correlations with the downstream areas. Moreover, all panels must have a spatial distance that corresponds to a level line.

### Modeling and analysis of Eyvashan Dam settlement using Plaxis2D software

During construction and the first impounding, the clay core of earth dams increases in pressure, leading to consolidation settlements in this area. The finite element method has the advantage of simplifying calculations by allowing analysis of earth dam settlements in two dimensions and under plane strain conditions. Plaxis2D software was utilized for the calculations of settlement values in this study. This software is written based on finite element relations and is capable of simulating the non-linear and time-dependent behavior of the soil as well as the analysis of the dam settlement during impounding and operation by considering the pore water pressure. This software allows for the modeling of dam construction layer by layer and the consolidation phenomenon. In this research, the largest cross-section of the dam (cross-Sect. 229), which has the largest number of instrumented sensors, has been used to model and predict the maximum settlement. The consolidation settlement model was created by considering the construction stages of the dam, leading to the embankment layering taking 8 stages and 1280 days. During the implementation process, the desired layer's soil is activated up to a height of around 10 m, and the weight of this layer is included in the displacement calculations at each stage. In Table 1, the parameters of the materials utilized in the settlement analysis are displayed.

**Table 1:** Parameters of materials used in numerical analysis.

Material	Mohr–Coulomb	Type material	E (Mpa)	g_dry_ (kN/m^3^)	g_wet_ (kN/m^3^)	g_sat_ (kN/m^3^)	C, C' (kPa)	
Core	Mohr–Coulomb	Un Drained Drained	35	17	20	21	63 28	11 24
Shell	Mohr–Coulomb	Drained	70	22.5	23.8	24.5	–	–
Filter	Mohr–Coulomb	Drained	45	19	21	22	–	–
Drain	Mohr–Coulomb	Drained	55	20.5	22	23	–	–
Alluvium	Mohr–Coulomb	Drained	500	21.5	**–**	23.2	–	–
Foundation	Mohr–Coulomb	Drained	5000	25	**–**	25.5	–	–
Cut off	Mohr–Coulomb	Drained	2500	24	**–**	24	–	–

**Figure 2:**
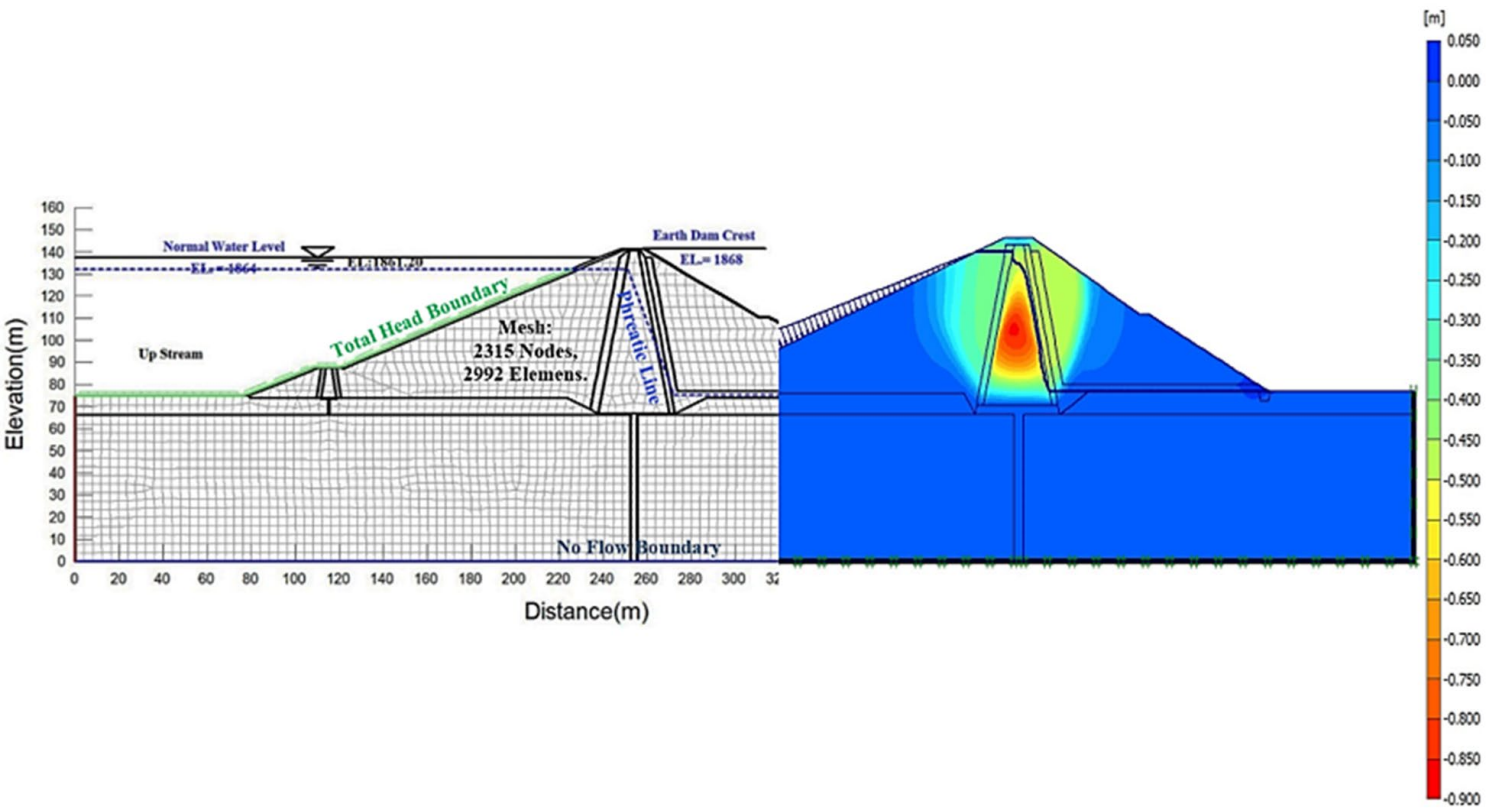
Deformed model and vertical displacement contours in Plaxis software.

Modeling the dam layer by layer and considering the construction time for each layer is due to the pore pressure trapped in the fine-grained and less permeable parts of the clay core, which first increases and then gradually decreases. The intensity and distribution of pore water pressure are highly dependent on the implementation process and not just on the consolidation characteristics and permeability of materials, but also on the drainage conditions. The pore pressure is only caused by the weight of the higher layers in a situation where the dam body strain is still insignificant because of drainage. The analysis was done by the effective stress method and triangular elements with 15-node linear strain were used to create the finite element model. The finite element network includes 2315 nodes and 2992 elements. Fine mesh is also used in this model (Fig. 2). Obviously, by choosing a finer mesh, the accuracy of the results will be higher, but the analysis speed will decrease.

behavior of the dam body and foundation materials. The Mohr–Coulomb model generally has 5 parameters, which are mainly known to geotechnical engineers, and these parameters can be obtained from common experiments. The first stage of the analysis pertains to implementing the first layer of the embankment, which was designed to adjust to actual conditions within 160 days. The consolidation phenomenon occurs in the lower layers as embankment layers are implemented, which is a fact. The performance of the analysis results in the creation of a distinct phase of consolidation analysis. A critical time model for consolidation analysis is calculated by the program automatically based on the permeability of selected materials and mesh dimensions. The program calculates less critical time when the mesh dimensions are smaller. In the same way, the second phase for the second layer of the embankment is analyzed and modeled for 320 days and until the end of the eighth phase equal to 1280 days. In this research, the static analysis of Eyvashan Dam was done by Plexis 2D software for the periods of construction, first impounding, and operation based on an actual period. Since the main purpose of this analysis is to compare the results of the numerical analysis with the results of the AI models, special cases such as rapid drawdown and earthquakes have not been investigated. Because Eyvashan Dam has not experienced any of these situations.

### Data collection and data pre-processing

The data set from the Eyvashan Dam settlement was utilized to make the predictions. At different levels, the dam settlement instrument data sets are included in these data sets. These data sets consist of multivariate time series that vary in terms of data and characteristics. SPSS and z-score methods were used for normalization in this research.

1 $$z-score=\frac{X-{mean}_{(X)}}{{Stdev}_{(X)}}$$

X is the calculated number, ${mean}_{(X)}$ is the mean of the set of calculated numbers and ${Stdev}_{(X)}$ is the standard deviation of the set numbers. Each element of the data set is transformed into a number with an average of 0 and a standard deviation of 1. The purpose of pre-processing is to eliminate any potential errors in the data. Some errors can occur when values are out of range, attributes are duplicated, and data is not defined in a suitable format for modeling. The data-preprocessing phase involves the selection of features, which is one of the most important activities. The process of selecting features involves entering those with the most information and the highest impact on decision-making and deleting unnecessary and unimportant features of the model. When using all features, especially when the number of features is high, it can lead to an increase in memory consumption and data inefficiency. Identification of imprecise data, duplicate data, data transformation, and discretization of continuous characteristic values are among the things that are included in the preprocessing phase.

### Clustering data for the settlement of Eyvashan Dam

To identify subgroups of observations in a dataset, clustering can be thought of as a diverse range of techniques. This method is unsupervised because there is no response variable. The goal of clustering observations is to have identical observations in one group and dissimilar observations in other groups. By clustering, we can identify observations that are similar and potentially group them into groups. This research utilizes K-means clustering and two-stage cluster analysis to divide a data set into k groups based on their simplicity and commonity.

### K-Means Cluster Analysis

**Figure 3:**
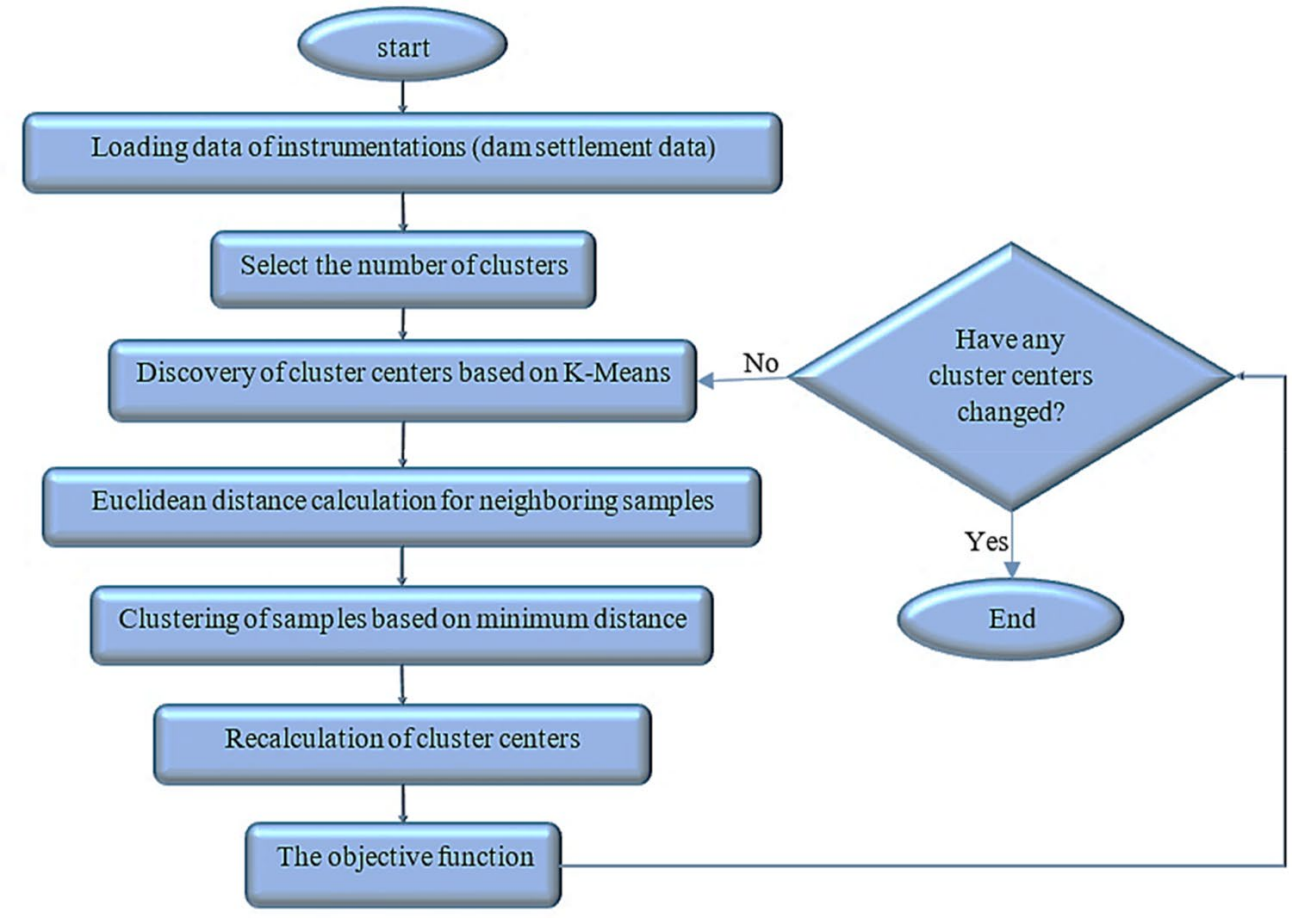
K-means clustering algorithm.

In this method, the data is divided into a predetermined number of clusters. The main idea is to define K centers for each cluster. The best choice for cluster centers in this algorithm is to place them as far as possible from each other. Next, each record in the dataset is assigned to the nearest cluster center. For this purpose, objective function optimization is used. The algorithm's results are heavily influenced by the initial guess about the centers of the clusters. The best option for cluster centers is to place centers at the greatest distance from each other. Finally, the dataset assigns every record in the cluster that is closest to it. Large data requires the use of the K-Means clustering algorithm. According to this method, the data is divided into a specific and well-known number of clusters. This method categorizes the respondents into several clusters assuming the number of clusters is known. The distance between two respondents is determined by using the Euclidean distance method in the K-Means clustering algorithm. The method begins by selecting the primary centers from the data. Using the method of the closest Euclidean distance to the cluster mean, each group's repeated observations are added to the cluster. The researcher needs to figure out the ideal number of clusters (K) as they proceed. Thus, the cluster centers change as the development progresses. As long as the average of the clusters does not change by more than a certain value or until we reach the repetition limit, this process will continue. K-means are typically created to lower the sum of squared errors within groups that are measured using Euclidean distances. This study used K-means clustering to randomly select the centers of 5 clusters based on their different characteristics. The average of each cluster has not changed, and the centers have not changed in the 21st iteration. Therefore, in this study, the clustering was completed after 22 steps. Figure 3 shows the K-means clustering algorithm.

### Group Method of Data Handling (GMDH)

**Figure 4:**
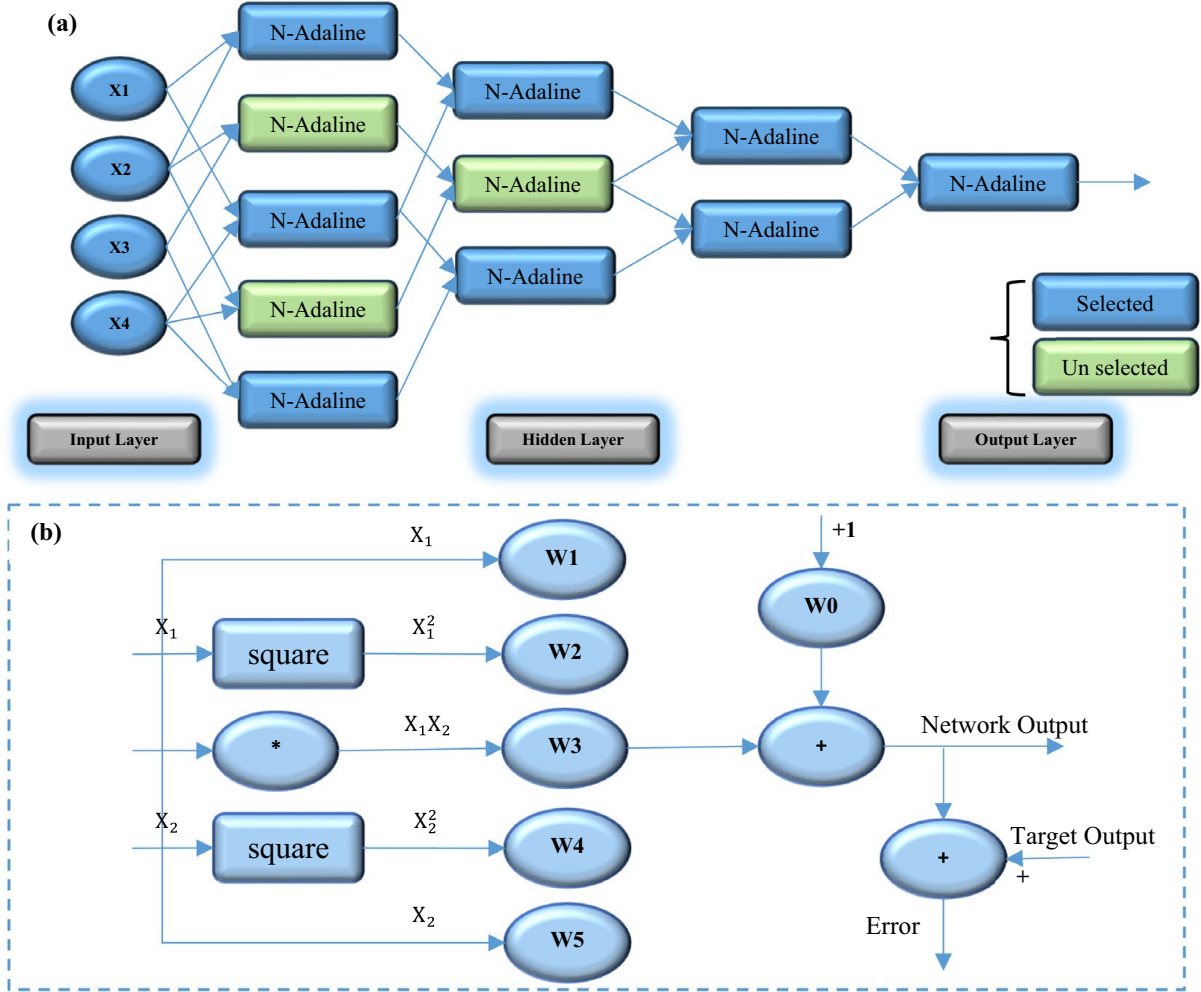
(**a**) Schematic of the structure of the group method of data handling (GMDH) model. (**b**) The structure of each N-Adaline.

Identifying, modeling, and predicting complex systems can be achieved using the GMDH method, which is one of the inductive approaches based on perception theory. Input layers, inner hidden layers, and output layers are present in the GMDH model, which is comparable to a multilayer neural network. Information is transferred from the input layer to the hidden layers based on their merit in the GMDH model and the multilayer neural network, but there is a difference between it and the multilayer neural network. Unlike the multilayer neural network, each neuron only receives two parameters. This means that the maximum number of neurons in the first layer can be equal to $\frac{n(n-1)}{2}$, where n is the number of input variables. The criterion for merit selection is the error rate of neurons. The schematic structure of the data group classification model is shown in Fig. 4. The initial layer can have a maximum of 6 neurons and five of them must be present in the formation of the second layer. Consequently, only the neurons that contribute to the development of the final structure of the GMDH model are displayed. A quadratic equation (Eq. 2) governs the function of neurons. The coefficients of these variables, including ${x}_{1}$, the input variables to the neuron, and wi, are determined in this equation when the GMDH model is trained.

2 $${Y}^{\sim }={w}_{0}+{w}_{1}{x}_{1}+{w}_{2}{x}_{2}+{w}_{3}{x}_{1}^{2}+{w}_{4}{x}_{2}^{2}+{w}_{5}{x}_{1}{x}_{2}$$

The GMDH model with several inputs and one output is a subset of the basis function components (Eq. 3):

3 $$Y\left({x}_{1},\dots ,{x}_{n}\right)={a}_{0}\sum_{i=1}^{m}({a}_{i}-{f}_{ij})$$

$f$ represents the initial function depends on different sets of inputs, $a$ represents the coefficients, and $m$ represents the number of basic functional components. Partial models or partial derivatives are used in the GMDH algorithm to find the optimal solution. The least squares method is used to estimate the coefficients of this model. The GMDH algorithm gradually increases the number of partial models and among them, finds a structural model with the desired complexity, which is represented by the value of at least one external criterion. This process is called the self-organized model. Therefore, it can be said that GMDH is a self-organized and unidirectional network that consists of several layers and each layer is formed from several neurons. The most popular basis function used in GMDH is the Kolmogorov–Gabor polynomial (Eq. 4).

4 $$y={a}_{0}+\sum_{i=1}^{m}\left({a}_{i}{x}_{i}\right)+\sum_{i=1}^{m}\sum_{j=1}^{m}\left({a}_{ij}{x}_{i}{x}_{j}\right)+\sum_{i=1}^{m}\sum_{j=1}^{m}\sum_{k=1}^{m}({a}_{ijk}{x}_{i}{x}_{j}{x}_{k})$$

This study aims to predict settlements based on the results of K-means clustering. The model of each settlement instrument a time t is based on upstream water level data, the piezometer data itself, and the data of two other piezometers in the same cluster. Equation (5) will be used to estimate the height of each piezometer in this scenario.

5 $${P}_{t}^{i}=f({P}_{t-1}^{i},{P}_{t-2}^{i},\dots ,{P}_{t-n}^{i},{P}_{t}^{j},{P}_{t-1}^{j},{P}_{t-2}^{j},\dots ,{P}_{t-o}^{j},{P}_{t}^{k},{P}_{t-1}^{k},{P}_{t-2}^{k},\dots ,{P}_{t-r}^{k},{h}_{t},{h}_{t-1},\dots ,{h}_{t-m})$$

### Random Forest (RF)

This method is based on a group of decision trees and group learning. The RF predictive model is based on averaging the results of all the relevant decision trees and performs classification with high accuracy for many data sets^17^. The RF algorithm can select the class with the most votes by voting and place it as the final class for classification. The most important feature of RF is its high performance in measuring the importance of variables and determining the role of each variable in predicting the response. If $\{h(X, \theta n)\}$ is the decision tree that forms the RF, then $\{\theta n\}$ is the number of independently distributed random vectors. All decision trees determine the final classification of the input vector $X$. It is necessary to first obtain n random vectors before building $n$ decision trees. These vectors θ1, θ2, θ3, ···, θn have the same distributions and are all independent. The decision tree is abbreviated as $hi(X)$ and the value of each $\theta i$ can be found using the formula $\{h(X, \theta i)\}$. If $(h1(X), h2(X), h3(X), \cdot \cdot \cdot , hn(X))$ are considered as $n$ classifiers and the random vectors $X$ and $Y$ are available, the corresponding function can be defined as Eq. (6) defined:

6 $$mg\left(X,Y\right)=a{v}_{n}\mathrm{\rm I}\left({h}_{n}\left(X\right)=Y\right)-{}_{j=Y}{}^{max}a{v}_{n}\mathrm{\rm I}({h}_{n}\left(X\right)=j)$$

$I (*)$ is an indicator function. The generalization error in $X$ and $Y$ space is:

7 $$P{E}^{*}={P}_{X,Y}(MG(X,Y)<0)$$

By increasing the decision tree, the generalization error $P{E}^{*}$ of the random vector $\theta i$ can be:

8 $$P{E}^{*}={P}_{X,Y}({P}_{\theta }\left(h\left(X,\theta \right)=Y\right)-{}_{j=Y}{}^{max}{P}_{\theta }(h\left(X,\uptheta \right)={\text{j}})<0)$$

**Figure 5:**
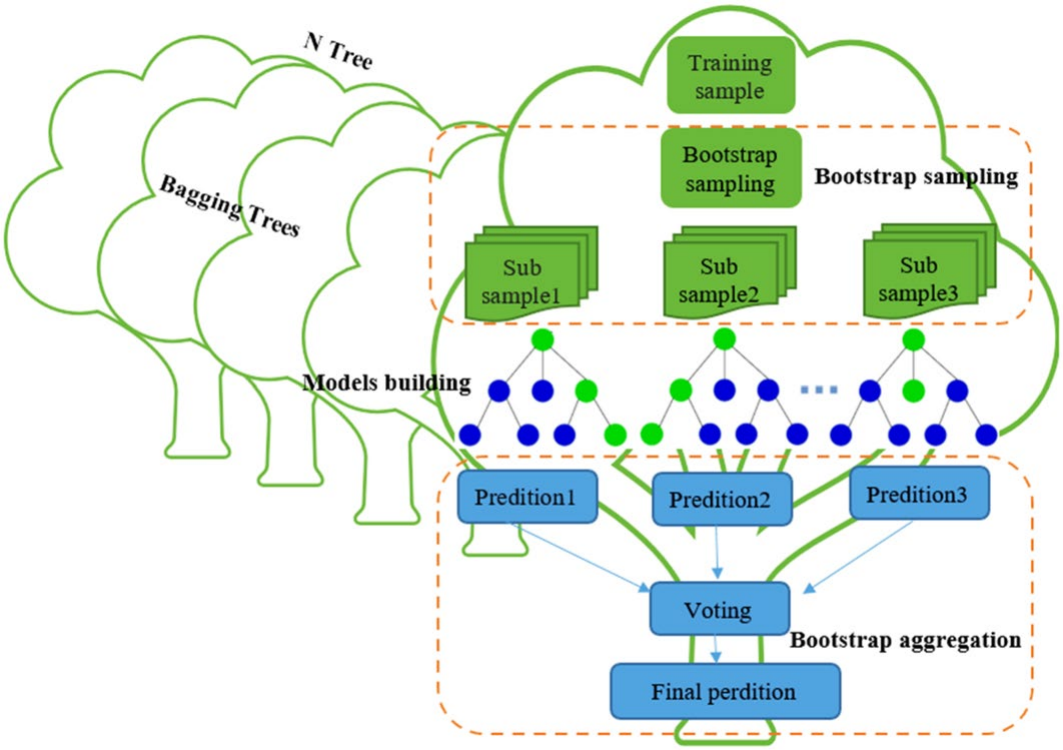
RF algorithm model.

Equation (8) shows that there will not be overfitting in the RF and the generalization error will be maximized as the number of decisions increases. The decision tree is constructed and combined using the classification method to fully ensure the stability of the learning process. Figure 5 shows the RF algorithm model.

### Multivariate adaptive regression splines (MARS)

The nonparametric MARS method is an adaptive regression method and works well for high-dimensional problems when the number of predictor variables is large. MARS is a soft computing technique that is made by combining several simple linear regression programs. Modeling in this method is based on the fitting of piecewise linear regressions, which are the simplest type of splines. The optimal MARS model is selected in a two-step process. In the first step, MARS builds an excessively large model through a formal mechanism, and in the second step, the basis functions that have the least contribution to the model are removed from the model until the optimal model is reached. The non-parametric and non-linear regression model is in the form of Eq. (9):

9 $${y}_{i}=f\left({x}_{i}1,{x}_{i}2,\dots ,{x}_{i}k\right)+{\varepsilon }_{i}$$

In Eq. (7), the regression function is a continuous function represented by f(x_i 1,x_i 2,…,x_i k) and ε_i is an estimate of the corresponding error. The model formulated using MARS to predict the output y is according to Eq. (10):

10 $$y={C}_{0}+\sum_{m=1}^{M}{C}_{m}{B}_{m}(x)$$

where M is the number of basic functions, ${C}_{0}$ is a constant value, ${B}_{m}$ is the mth basic function and ${C}_{m}$ is the corresponding coefficient of ${B}_{m}(x)$. $f$ is a set of basis functions (BFs). Basis functions are spline functions that are generally expressed as polynomial functions. Summary of the basic function equations for the qth order lines are presented as Eqs. (11) and (12):

11 $${\left[-(x-t)\right]}_{+}^{q}=\left\{\begin{array}{ll}{(t-x)}^{q} &\quad if x<t\\ 0 &\quad otherwise\end{array}\right.$$

12 $${\left[+(x-t)\right]}_{+}^{q}=\left\{\begin{array}{ll}{(t-x)}^{q}&\quad if x\ge t\\ 0 &\quad otherwise\end{array}\right.$$

where $t$ is the cutting position, which is called a node, (q $\ge 0$) is the power of the spline functions and indicates the degree of smoothness of the splines, and the subscript $+$ indicates a positive value.

### Performance evaluation of AI models

The purpose of the validation of models is to measure their accuracy. To accomplish this task, various statistics are compiled and applied. In this research, to evaluate the models, Determination coefficient (DC) and Root mean square errors (RMSE) have been used, the value of which should be in the desired range in both training and testing stages (DC ≃ 1, RMSE ≃ 0). Equations (13) and (14) are used to calculate the value of the statistics mentioned^18,19^.

13 $${\text{RMSE}}=\sqrt{\frac{1}{{\text{N}}}(\sum_{{\text{i}}=1}^{{\text{N}}}{\left({y}_{{obs}_{i}}-{y}_{{com}_{i}}\right)}^{2}}$$

14 $$DC=1-\frac{\sum_{i=1}^{N}{({y}_{{obs}_{i}}-{y}_{{com}_{i}})}^{2}}{\sum_{i=1}^{N}{({{y}_{obs}}_{i}-{\overline{y} }_{obs})}^{2}}$$

In these relationships, ${y}_{com}$ is the data related to the model results, ${y}_{obs}$ is the observational data (accurate instrument), ${\overline{y} }_{obs}$ is the average of the observational data, and N is the number of data.

## Application

### Engineering example and data

**Figure 6:**
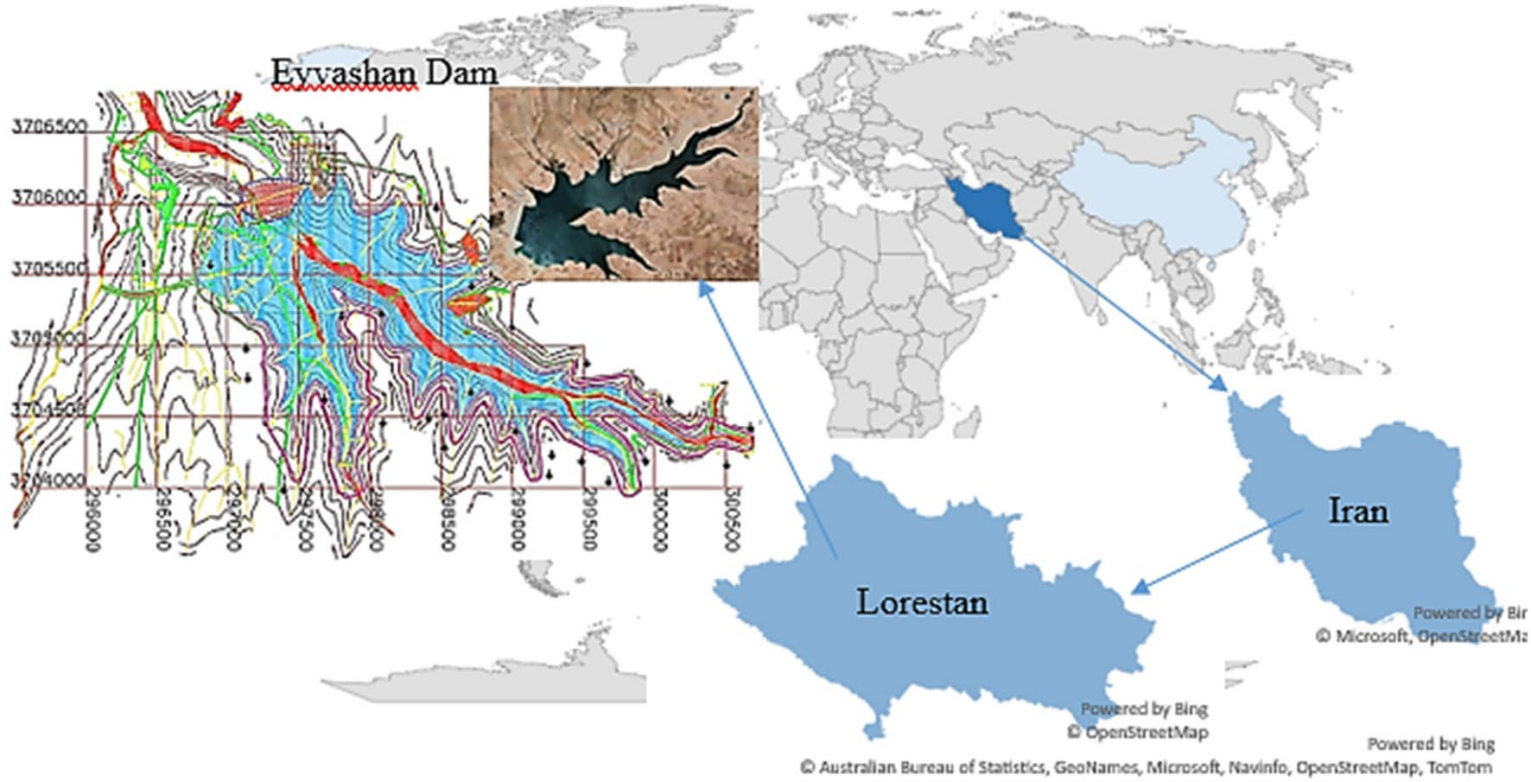
The geographical location of Eyvashan Dam^20^.

Eyvashan Earth Dam is located in the east of Khorramabad city in the enclosed plain between Razan and Zagheh passes. From the geographical point of view, the location of the Eyvashan Dam on located in the geographical coordinates of 48° 49′ 2″ east longitude and 33° 28′ 31″ north latitude (Fig. 6).

**Figure 7:**
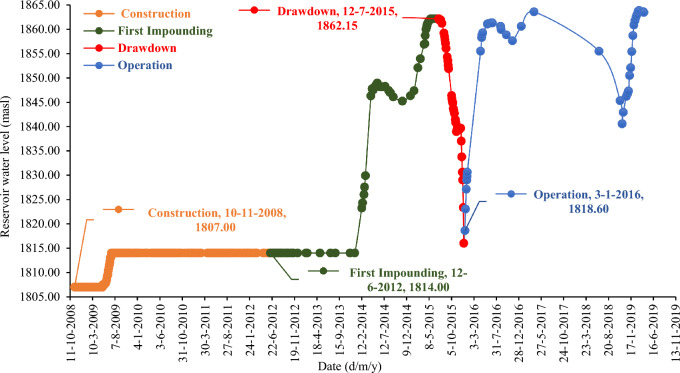
Changes in the water level of the Eyvashan Dam.

At the beginning of 2012, the executive operation of the construction of the Eyvashan Dam began. After a delay of about 30 months, in the middle of 2014, the executive operation of the dam was started again, and finally, the embankment operation of the dam body was completed in June 2015. At the end of 2013, the dam was officially impounded. Changes in the water level of the Eyvashan Dam reservoir in different phases are shown in Fig. 7.

### Instrumentation sections in Eyvashan Dam

**Figure 8:**
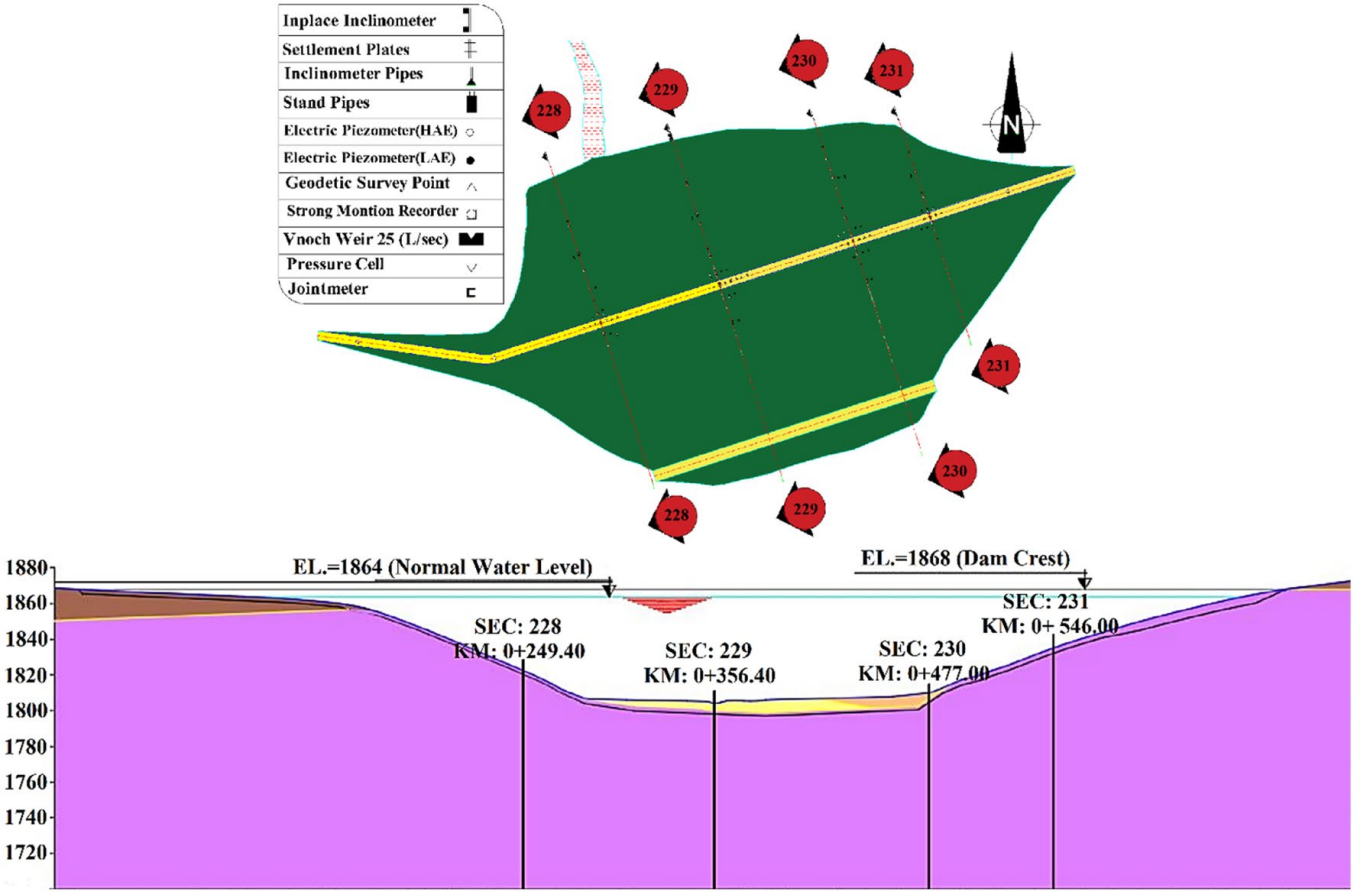
Eyvashan Dam instrumentation plan and cross-section.

The Eyvashan dam's instrumentation is divided into four cross-sections corresponding to numbers 228, 229, 230–230, and 231 at 249, 356, 477, and 546 km (Fig. 8).

**Figure 9:**
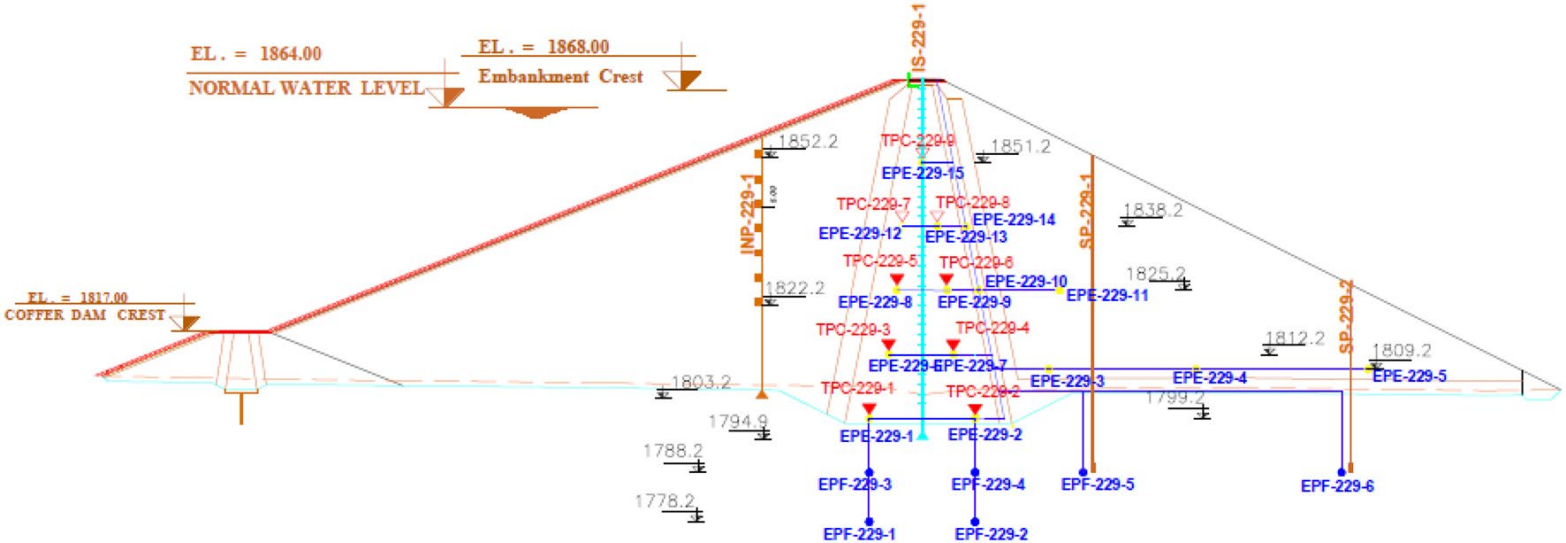
Placement of Instrumentation of Eyvashan Dam at cross-section 229.

The instrument installed in Sec-229 of the Eyvashan Dam has been analyzed in this study to determine its behavior. Figure 9 shows Sec-229 of the instrumentation of the Eyvashan Dam. The highest level of instrumentation relates to Sec-229 with 7 levels and the least number of levels of instrumentation is related to section 231 with 5 levels.

### Monitoring results of instrumentations of Eyvashan Dam

**Figure 10:**
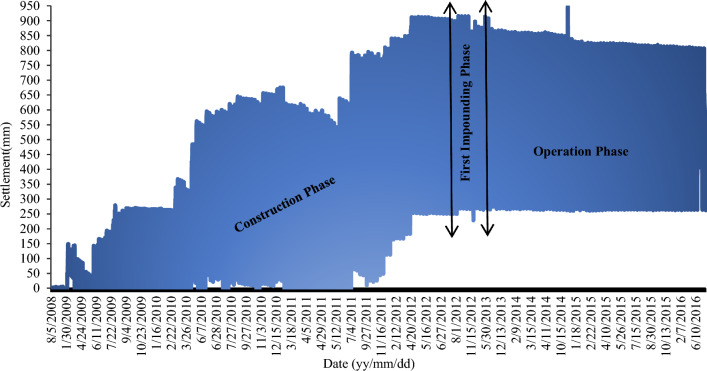
The settlement of Eyvashan Dam in phases of construction, first impounding, and operation (IS229).

**Figure 11:**
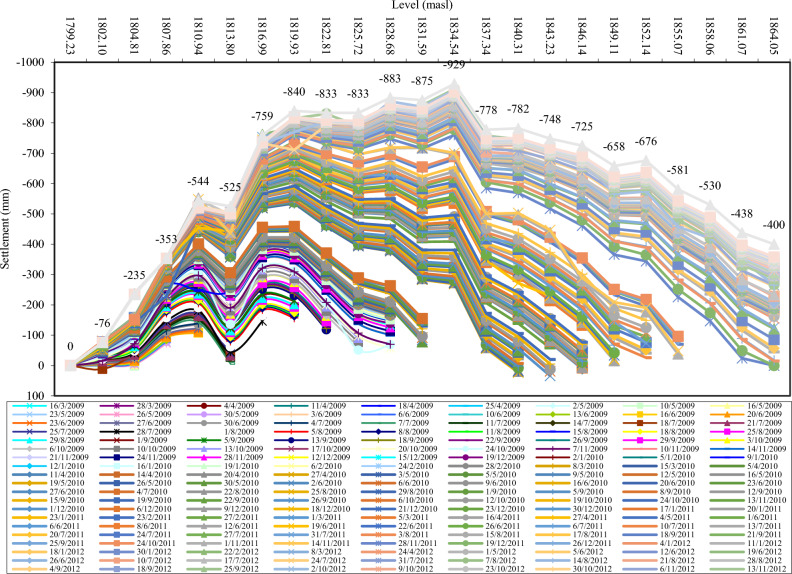
Changes of settlement compared to height in Eyvashan Dam (IS229).

**Figure 12:**
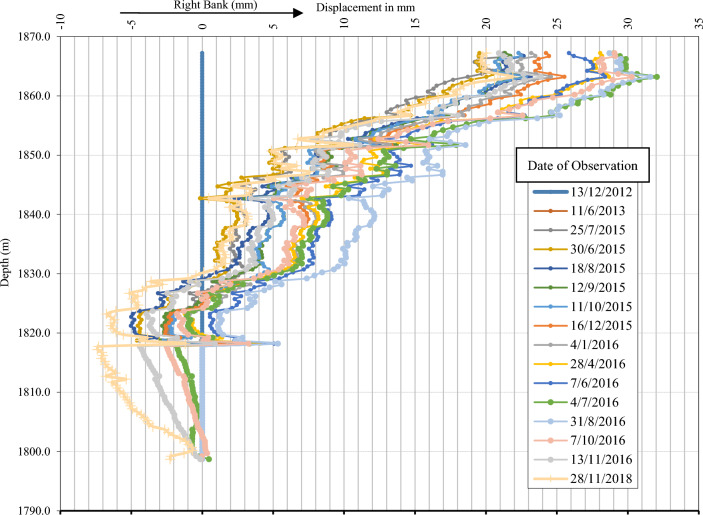
Horizontal displacement changes of Euvashan Dam (Sec 229).

The clay core has a settlement meter that records the settlement of Eyvashan Dam. The amount of settlement in the IS229 instrument is measured using 22 magnets installed at regular intervals. To investigate the changes in the settlement, the results of the instrumentations were used from the beginning of the executive operation of the construction of the dam body to the period of the water impounding and operation. The amount of settlement is higher in the middle part (due to the greater depth of the material that can be settled) and it has lower values in the sides. Therefore, the maximum settlement of the dam core occurred in the last reading and at the end of the construction phase in the middle levels (1835 masl) in the amount of 809 mm. In the upper third and near the crest of the dam (level 1864–1835), the amount of calculated settlement varies from 600 to 300 mm, and this amount has decreased to 200 mm in the lower third (level 1810) (Figs. 10 and 11). At the height of the upper third, the direction of the horizontal movement of the dam fluctuates from upstream to downstream. The maximum horizontal displacement in Evashan Dam is 32.6 mm (Fig. 12). The upstream shell materials tend to collapse and settle during the first period of impounding of the reservoir due to waterlogging. Since the lower layers of the crust are usually denser than the upper layers, therefore, at the end of the construction stage and after the first impounding of the dam reservoir, most movements due to the collapse may occur in the upper parts of the dam.

## Results and discussion

The settlement results of the Eyvashan Dam show that the settlement of the dam core changes continuously and gradually after the start of construction due to the embankment operation and the creation of additional load on the previous layers. It was found that the stoppage of embankment operations during the construction period is related to the uneven increase of core settlement due to various reasons. The majority of settlements during construction are made up of instantaneous or elastic settlements. The elasticity of materials results in instantaneous settlements when new layers are loaded on older layers within a short period. Settlements are influenced by the drainage rate of pore water pressures created during dam construction, which is why consolidation occurs over the long term. Significant consolidation settlements cannot form due to the high permeability of earth dam shells. Also, due to the low permeability of the clay core, drainage of pore pressure from the core during construction is carried out for a long time. As a result, the clay core consolidation settlement is more than the shell. Typically, the initial settlement for the dam is significantly greater than the consolidation settlement, which eventually decreases and reaches a constant value. Considering that at the end of the construction and after the first impounding of the dam, the lower layers are usually denser than the upper layers, most movements due to settlement occur in the upper parts and near the crest of the dam. As a result, there have been a small amount of changes made to the dam settlement during this period. When impounding begins, the settlement is toward the bottom of the core, and with the passage of time and the increase of the water level, the intensity of the settlement of the core is reduced, and slight movements towards the crest are seen. During the operation phase, the pattern of settlement values remains consistent. Maintaining dam stability becomes more important as settlements increase. The construction process of the dam necessitates the repair of damages caused by inhomogeneous settlement due to the minimal or zero water volume of the lake. Because the settlements in a given point are a function of the height of the soil on it (overhead) and the height of the embankment, therefore, most of the settlements occur almost at the middle level of the dam. An ANN algorithm has been used to select a stable feature and remove inappropriate and redundant features, which can predict complex and non-linear relationships and extract effective features for modeling the target variable. Therefore, the monitoring data is divided into two categories: training (80%) and test (20%), and to calculate the model error, the weighted average of the training and test errors in the sensor of the studied dam settlement instrumentation. RMSE was chosen as the error index. Because in the feature selection process, the goal is to identify more important and effective features than its estimation on the output variable. Therefore, the error of the training data is more important than the error of the test data, and the relationship between the input variables and the target is obtained according to the training data. To increase the efficiency of network training, first, all the data were normalized between 0.1 and 0.9. Then the sensitivity analysis was performed by Hill's method and the influence of the input parameters on the output parameter (settlement) was determined. Therefore, in this work, embankment level (m.a.s.l), water level (m.a.s.l), consolidation time (day), and embankment rate (m.a.s.l/day), were identified as effective features in the clustering of Eyvashan Dam settlement. According to the ANOVA table in the k-means method, each input played an appropriate role in the recovery and separation of clusters, which was an acceptable criterion for clustering. The greater the F value in the variance analysis, the more important the variable is in separating clusters from each other. The embankment level, consolidation time, water level, and embankment rate were all significant factors when separating clusters using the K-means method. Table 2 shows the variance analysis for clustering using the k-means method.

**Table 2:** Variance analysis of clustering using the K-means method.

Parameter	Cluster		Error		F	Sig
	Mean Square	df	Mean Square	df		
Embankment Level (m.a.s.l)	15.185	4	.126	65	120.406	.000
Consolidation time (day)	14.621	4	.162	65	90.398	.000
Water Level (m.a.s.l)	13.458	4	.233	65	57.688	.000
Embankment Rate (day)	11.062	4	.381	65	29.073	.000

**Figure 13:**
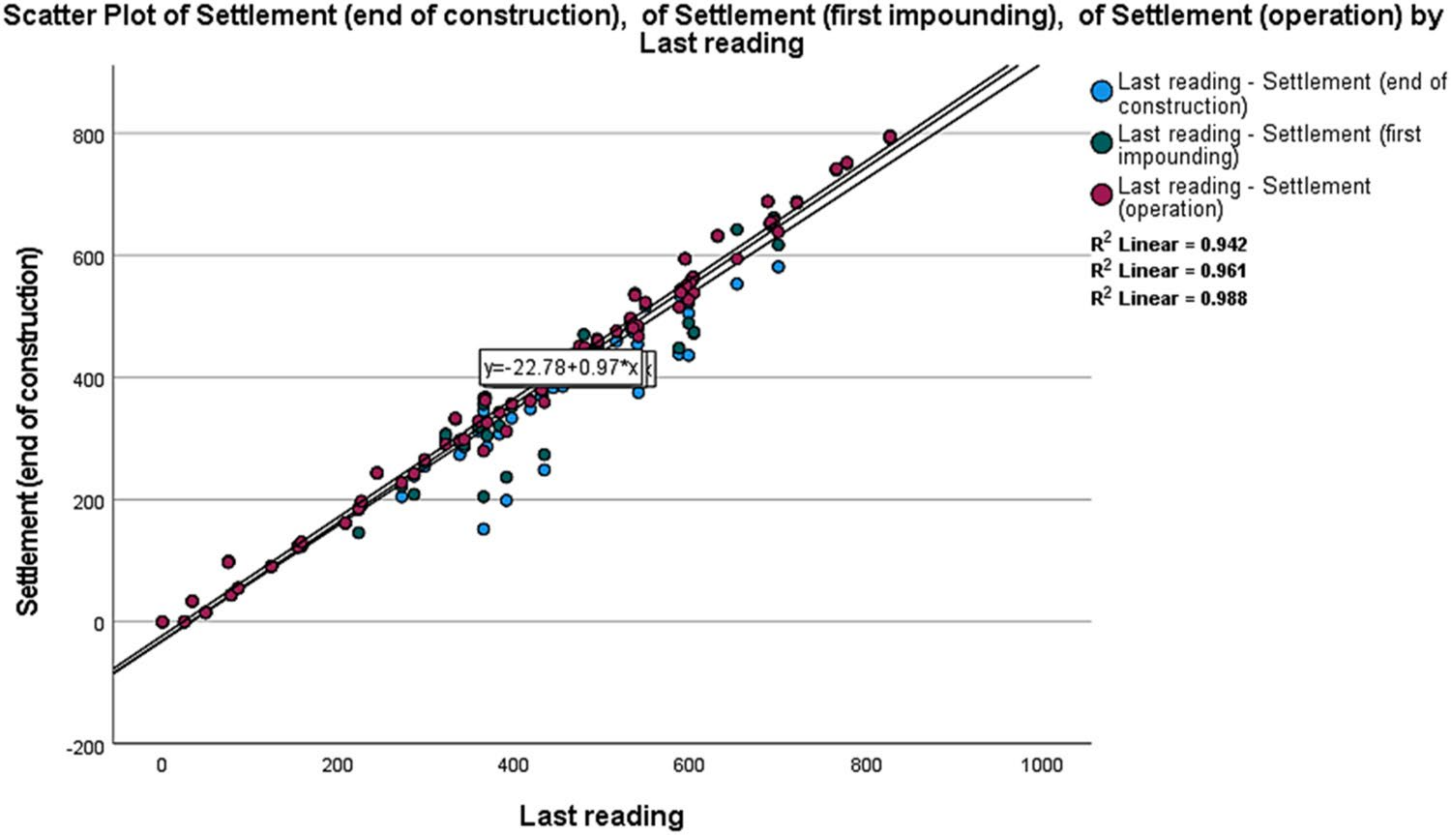
K-means analysis and settlement data correlation rate.

The analysis shows that the first group has 6 participants, the second group has 20 participants, the third group has 12 participants, the fourth group has 24 participants, and finally, the fifth group has 7 participants. The connection between the three stages of construction's end, which are the first impounding and operation during the last reading of the settlement, is illustrated in Fig. 13.

**Figure 14:**
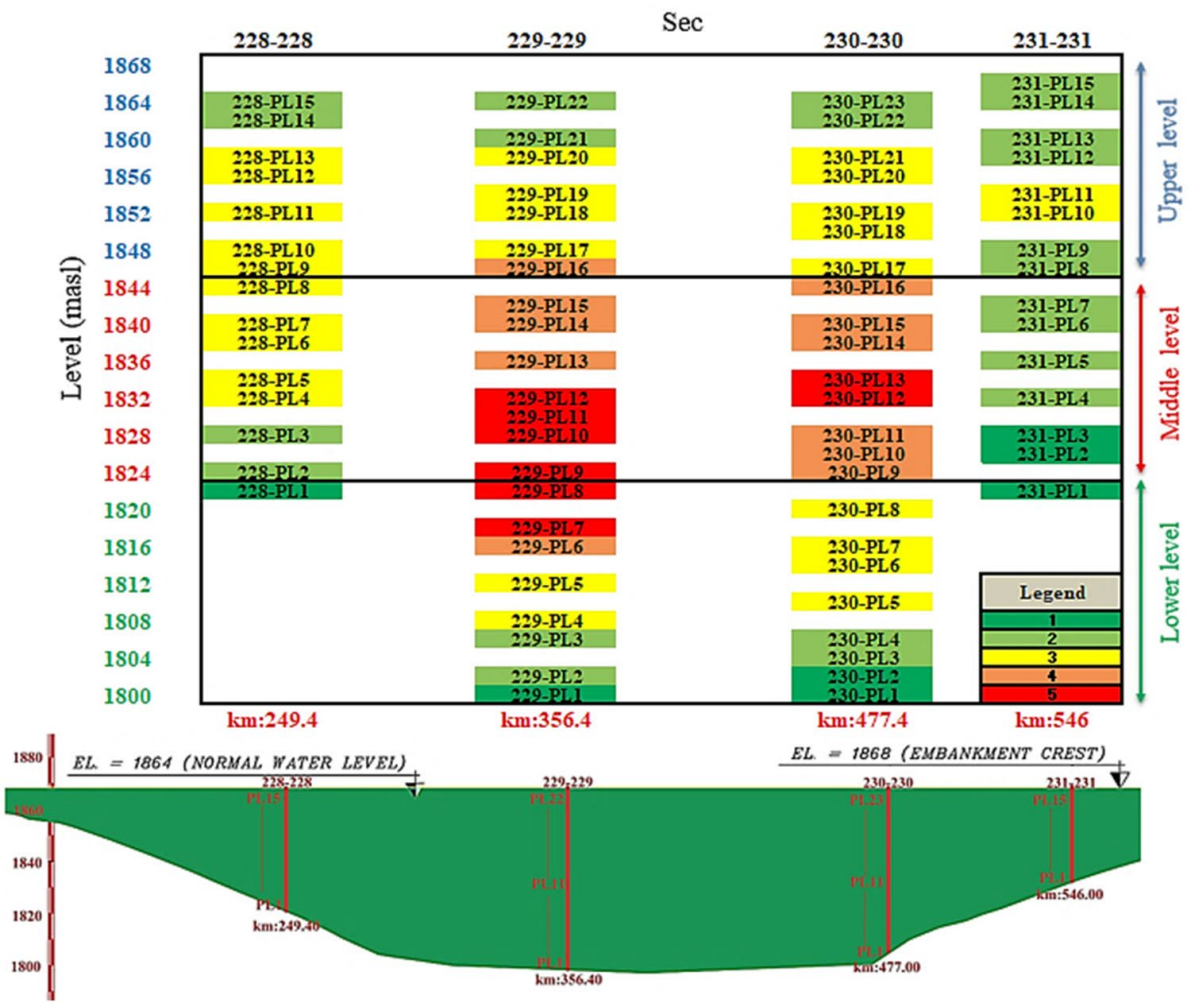
Clustering of Eyvashan Dam settlement by k-means method.

In Fig. 14, the results of k-means clustering are displayed.

The height and level of the embankment (overhead) results in settlements usually occurring near the middle of the dam. The results show that over time the compressibility and instant consolidation of the clay core increases from low to high levels in the core of the earthen dam and causes an increase in settlement. Additionally, there has been a decrease in the amount of overhead that contributes to the dam's density and settlement. The dam's core has a nearly balanced interaction between these two effects on its middle levels. The general trend is fairly close to that of the instrumentation results, with the clustering and numerical analysis results agreeing relatively well. Differences in geotechnical parameter values between the model and implementation may lead to a discrepancy between the numerical analysis and measured values. The core's maximum settlement has been determined by the clustering models and numerical analysis, taking into account the results of the instruments at the middle levels.

### Estimation of dam settlement using AI models

The settlement can be predicted by using the results of five clusters with higher correlations. To model settlement in other settlement meter plates of the cluster, we used the upper, middle, and lower levels of the dam, taking into account the same clustering. Other cross-sections in the same cluster have been tapped into for information and data. The PL230-20 plate is being modeled at the upper level of the dam using the results of the PL228-12, PL229-19, and PL231-11 plates as per this scenario. In the middle part, plate PL229-9 was modeled with plate numbers PL229-12, PL230-13, and PL229-8 in the same way. To predict and model the settlement in plate PL228-1, plates PL231-1, PL229-1, and PL230-1 were utilized in the lower part of the dam. Time delay approaches were considered in the GMDH model. The GMDH model and Plaxis numerical model were used to estimate settlement modeling, as presented in Table 3. The results of the AI models with four input features including embankment level (m.a.s.l), water level (m.a.s.l), consolidation time (day), embankment rate (m.a.s.l/day), and the numerical model were compared with the aforementioned statistical indicators.

**Table 3:** Comparison of numerical and AI models in estimating the settlement.

Model	PL230-20				PL229-9				PL228-1			
	Test		Training		Test		Training		Test		Training	
	RMSE	DC	RMSE	DC	RMSE	DC	RMSE	DC	RMSE	DC	RMSE	DC
GMDH	1.6947	0.9837	1.8635	0.9965	1.3121	0.9899	1.3915	0.9915	1.9532	0.9786	2.0230	0.9843
RF	1.6523	0.9625	1.7252	0.9857	1.4264	0.9762	1.5710	0.9803	1.8560	0.9568	1.8233	0.9695
MARS	1.9234	0.9533	2.0190	0.9716	1.5123	0.9655	1.6231	0.9774	2.0287	0.9412	2.7906	0.9601
FEM-Plaxis	1.8589	0.9568	1.9355	0.9632	1.5994	0.9602	1.6745	0.9699	2.8671	0.9471	3.0756	0.9585

**Figure 15:**
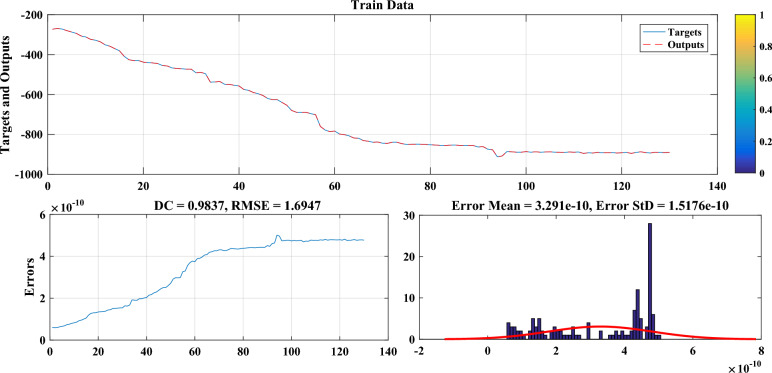
Calculated and observed settlement values during the test period.

As can be deduced from Table 3, DC and RMSE values in all AI models perform better than the numerical model. Also, the GMDH model shows a better performance in settlement modeling with 0.9837 and 1.6947, respectively, compared to other AI models used in this study. Figure 15 shows the performance of the GMDH model for clustering dam settlement modeling.

The Eyvashan Dam's settlement can be accurately modeled and predicted by this model, as demonstrated by the GMDH method.

## Conclusion

The purpose of this article is to use the information on instrumentation to identify and control the settlement areas in the core of Eyvashan Dam, which is monitored by a numerical analysis model and spatiotemporal clustering model. Investigating the process of changes in the settlement of the dam core using instrumentation monitoring is such that the settlement occurs at a faster rate at the beginning and in the construction phase, and then at the end of construction and the start of operation, the rate of settlement decreases and reaches a constant value. According to the findings, the dam's settlement was more than 90% completed before impounding, followed by secondary consolidation settlement. The first period of impounding causes an increase in the amount of settlement in the higher levels compared to the lower levels of the dam. This problem is due to the lack of enough time to consolidate at higher levels. The dam body's settlement changes are parabolic in shape, and the highest settlement (809 mm) happened near the center of the core. The settlement is within the acceptable range for settling, which is equal to 1.2% of the dam's height. Since the maximum horizontal displacement is only 32.6 mm, there won't be any harm done to the dam. The spatiotemporal model presented can accurately partition the settlement area and analyze the dam's behavior during different periods, as evidenced by the results of this research. The spatiotemporal clustering method was highlighted through the presentation of contour maps. The k-means analysis method's reliability and delineation of settlement zoning conditions are demonstrated by the correlation between the results presented in the clustering method. Using spatiotemporal clustering results, the AI models were utilized to predict the dam's settlement. According to the testing and training section, the accuracy of the proposed model is acceptable for predicting all AI models and the performance of the GMDH model in predicting the settlement is better than other used models. The high generalizability of the GMDH approach was attributed to its self-organizing nature. According to the obtained results, the proposed two-stage model including K-means cluster analysis clusterin and AI models can predict the settlement of earth dams more accurately.
